# Making Time for Nature: Visual Exposure to Natural Environments Lengthens Subjective Time Perception and Reduces Impulsivity

**DOI:** 10.1371/journal.pone.0141030

**Published:** 2015-11-11

**Authors:** Meredith S. Berry, Meredith A. Repke, Norma P. Nickerson, Lucian G. Conway, Amy L. Odum, Kerry E. Jordan

**Affiliations:** 1 Department of Society and Conservation, University of Montana, Missoula, Montana United States of America; 2 Department of Psychology, University of Montana, Missoula, Montana, United States of America; 3 Department of Psychology, Utah State University, Logan, Utah, United States of America; VU University Medical Center, NETHERLANDS

## Abstract

Impulsivity in delay discounting is associated with maladaptive behaviors such as overeating and drug and alcohol abuse. Researchers have recently noted that delay discounting, even when measured by a brief laboratory task, may be the best predictor of human health related behaviors (e.g., exercise) currently available. Identifying techniques to decrease impulsivity in delay discounting, therefore, could help improve decision-making on a global scale. Visual exposure to natural environments is one recent approach shown to decrease impulsive decision-making in a delay discounting task, although the mechanism driving this result is currently unknown. The present experiment was thus designed to evaluate not only whether visual exposure to natural (mountains, lakes) relative to built (buildings, cities) environments resulted in less impulsivity, but also whether this exposure influenced time perception. Participants were randomly assigned to either a natural environment condition or a built environment condition. Participants viewed photographs of either natural scenes or built scenes before and during a delay discounting task in which they made choices about receiving immediate or delayed hypothetical monetary outcomes. Participants also completed an interval bisection task in which natural or built stimuli were judged as relatively longer or shorter presentation durations. Following the delay discounting and interval bisection tasks, additional measures of time perception were administered, including how many minutes participants thought had passed during the session and a scale measurement of whether time "flew" or "dragged" during the session. Participants exposed to natural as opposed to built scenes were less impulsive and also reported longer subjective session times, although no differences across groups were revealed with the interval bisection task. These results are the first to suggest that decreased impulsivity from exposure to natural as opposed to built environments may be related to lengthened time perception.

## Introduction

Numerous and pressing environmental issues (e.g., species extinction, accelerated climate change, natural resource exploitation, overpopulation) result from poor human decisions [1,2]. For example, despite climate scientists’ description of current emissions as "dangerous to extremely dangerous" [3], anthropogenic influenced global carbon emissions have surpassed even the worst scenarios predicted by the Intergovernmental Panel on Climate Change [4]. Similarly, despite the necessity of biological diversity for long-term human and ecosystem health, medical research, and combating infectious disease [1], biodiversity is decreasing as extinction rates of microbial, plant, invertebrate and vertebrate species continue to increase across ecosystems [5,6].

The repercussions of poor human decision-making extend beyond environmental issues. Poor human decision-making also underlies many grave societal dilemmas such as drug addiction and obesity [7]. The National Institute on Drug Abuse (NIDA) estimates that the United States spends $600 billion dollars annually on drug and alcohol related issues including crime, health care, and losses in work productivity [8]. Arguably equally detrimental is the current rise in obesity, from which over one third of Americans (over 100 million people) now suffer [9]. Environmental and global health issues are far from mutually exclusive (see [1, 2] for discussion). Although often complex in nature, these issues highlight the need for more detailed understanding concerning influences of human behavior across numerous decision-making processes.

### Impulsivity and Decision-Making

One critical factor driving human decisions concerning natural resources and other societal issues is the appeal of immediate payoffs. The tendency to choose small but immediate outcomes over larger delayed outcomes is largely considered a form of impulsivity. Impulsivity is a multifaceted construct that can be measured in different ways [10–14] and has implications for environmental [2,11], individual [7] and societal decision-making processes [11,12,15]. Impulsivity can be conceptualized as the inability to delay gratification, and is often associated with the choice of a smaller immediate reward over a larger delayed reward. A common way of measuring impulsivity is delay discounting, which describes the decrease in value of an outcome or reward with the increase in delay to its receipt [10].

Delay discounting has garnered much attention recently due to its generality and predictive validity [16,17] across a range of maladaptive behaviors, including drug and alcohol addiction [18–20], obesity [21], and problematic gambling [22]. Beyond addictive behaviors, Chabris and colleagues [23] have suggested that degree of delay discounting is the single most important predictor of general real world behavior currently available, even when measured by a brief laboratory task. In other words, those who are impulsive within a laboratory delay discounting task using either real or hypothetical rewards [24] tend to also be impulsive in real world situations—potentially representing a relatively enduring trait [17], driven by genetic [25], neurocognitive [26,27], and environmental [28,29] influences.

New research also suggests that reducing degree of delay discounting in one realm may also decrease degree of delay discounting in other realms [30,31]. Decreasing impulsivity in delay discounting, therefore, may be representative of global reductions in impulsivity across numerous behaviors. For this reason, current research has focused on delay discounting in general, and techniques that reduce delay discounting more specifically ([32,33]; for a review see [34]).

### Can Nature Reduce Impulsivity?

One recent line of research has shown that people discount delayed rewards less (i.e., are less impulsive/more self-controlled) when visually exposed to natural environments such as forests or mountains [2,35]. For example, Berry et al. [35] showed that individuals exposed to photographs of natural environments exhibited significantly less impulsivity in a delay discounting task than those exposed to photographs of built environments. Using a different monetary discounting task, van der Wal and colleagues [2] also found that visual exposure to natural photographs on a computer screen resulted in less impulsivity than built photographs. In a follow-up experiment, similar results were obtained when participants walked through either natural landscape environments or built landscape environments and then chose between receiving money now or in the future.

Why does exposure to natural environments result in more self-control? One idea suggested by van der Wal et al. [2] involves evolutionary processes: Pictures of natural environments signal resource abundance and therefore individuals discount future outcomes less. Pictures of built environments may signal competition for mates and resources—an environment where impulsivity (i.e., the choice of smaller but immediate outcome) may be more beneficial.

A potentially complementary mechanism by which natural environments might increase self-control involves time perception. It is possible that restorative natural environments are tranquil—reducing general arousal, and/or increasing attentional capacity—and that viewing them lengthens the perception of time. Preliminary evidence exists supporting the idea that exposure to nature lengthens time perception, and a growing body of evidence has shown that lengthening time perception through various means decreases impulsivity. Rudd, Vohs, and Aaker [36] showed that scenes inspiring *awe* (which included, but were not limited to, nature scenes) caused people to feel they generally had more time available. However, this prior research only measured perceptions of future time availability and did not measure whether natural environments psychologically slow down time as it is actually occurring. Thus, it remains to be seen whether there is a direct nature-time estimate connection.

Several studies have demonstrated associations between altered sense of time perception and impulsivity [37–41]. For example, Wittman and colleagues [42] have identified neural substrates implicated in time perception and estimation, and impulsive decision-making (e.g., striatum activation). Based on these findings and physiological time perception mechanisms such as circadian and circannual rhythms, Wittman and Paulus [43] propose two time perception mechanisms that are biologically and culturally determined and that subsequently impact impulsive decision-making. Furthermore, independent of any experimental manipulation, those who estimate time of stimulus presentation to pass more slowly on an interval bisection task show less impulsivity on a delay discounting task ([13], see also [34] for a review including time saliency and perception influences on impulsivity). These findings show that time perception and time saliency are related to impulsivity in delay discounting, and that impulsivity might be influenced through time perception. Taken together, these studies suggest that viewing nature may lengthen time perception, consequently reducing impulsivity and benefiting human decision-making.

### The Present Study

No prior research specifically investigates possible effects of natural environments on time perception, nor has any prior research tested the effect of natural environments on both impulsivity and time perception in the same study. The present study aims to fill this gap by administering measures of delay discounting and time perception after visual exposure to natural versus built environments. Specifically, we hypothesized viewing scenes of natural environments would result in (1) less impulsivity and (2) lengthened time perception, relative to viewing scenes of built environments. We also expected that (3) lengthened time perception would be inversely related to impulsivity. If exposure to natural scenes reduces impulsivity and lengthens time perception, this will provide initial insight into possible mechanisms driving reduced impulsivity with exposure to natural scenes.

## Method

### Participants

Forty-five undergraduate students were recruited from an introductory psychology course.

### Ethics Statement

Participants provided their written informed consent and received course credit for participation. Individuals under the age of 18 were not permitted to participate in this study. The University of Montana Institutional Review Board approved all experimental and consent procedures.

### Setting and Apparatus

Participants were tested individually in a quiet room equipped with a desk, chair, computer and mouse. There were no cues related to time visible on the computer during the experimental sessions and participants did not have watches or cellular phones on their person. Experimental manipulations and data recording were programmed using E-Prime 2.0®. Survey and demographic data were collected using Qualtrics®.

### Stimuli

The stimuli used in the present study have been used previously to test differences in attention restoration across natural and built environments [44], as well as differences in impulsivity in a delay discounting task across natural and built environments [35]. In the natural condition, participants viewed photographs of nature (e.g., mountains, forests, lakes). In the built condition, participants viewed photographs of built environments (e.g., buildings, cities, roads).

### Procedure

Participants were assigned by block randomization to either the natural condition or the built condition. Within each condition, two separate tasks were completed. One task (the delay discounting task) was used as the measure of impulsivity. The other task (the interval bisection task) was used as a measure of short interval time perception. The order of these two tasks was randomly assigned across participants. In order to demarcate the tasks, the program for each was started by the research assistant [45]. Following the completion of each task, two long interval measures of time perception were collected (described in detail below), followed by collection of basic demographic information.

#### Delay Discounting Task

Instructions were provided on the computer screen which led the participants through the task, and also noted that they should choose whichever options they preferred [46]. Participants used the mouse to progress through instructional screens and to make their choices. Before the start of the delay discounting task and between each delay block, participants viewed either natural or built photographs. Photographs were displayed for 10-s each. Prior to the delay discounting task participants viewed 25 photographs, and between each delay block viewed 5 photographs (which were randomly selected from the original set of 25). All aspects of the experiment were identical across conditions with the exception of the condition-specific photographs.

Participants were tested in the delay discounting task using hypothetical monetary outcomes. All choice screens presented the wording "Would you rather have [amount] now or [amount] in [delay]?” Participants selected the immediate or delayed outcome to progress, and the side of immediate or delayed amount varied randomly across trials. The mouse cursor automatically centered after each choice. Participants completed 10 practice trials that were designed to familiarize the participant with the immediate and delayed tradeoff options. Following the 10 practice trials and condition-specific stimuli exposure, all participants experienced the titrating amount discounting procedure described below. Delays tested were 1 day, 1 week, 1 month, 6 months, 1 year, 5 years, and 25 years, in that order. Thus, there were 7 delay blocks with 5 photographs presented between each.

For each trial in the titrating amount delay discounting procedure, participants chose between immediate and delayed options. The first trial at each delay began with the choice of $50 now or $100 after a delay, and the immediate amount increased or decreased based on the participant’s response with each subsequent trial. If the immediate outcome was selected, the amount of the next immediate outcome decreased; if the delayed outcome was selected, the amount of the next immediate outcome increased (see [46] for more detail on the titration procedure).

#### Short Interval Timing Task: Temporal Bisection

In the interval bisection task, participants categorized whether the condition-specific stimuli were presented for a duration closer to 0.4 (i.e., short anchor) or 1.6 (i.e., long anchor) seconds across a range of durations (see [13] for a detailed description of an interval bisection task and analyses). These durations were selected to evaluate whether differences in time estimates using relatively short durations of stimulus presentation (i.e., milliseconds/seconds) would result from viewing natural versus built photographs. Using these durations of stimulus presentation, no differences in time estimations were revealed between natural or built conditions, and no relations to impulsivity were found. Therefore, the task is not described further. While these null data are not discussed further here, this topic is revisited in the discussion as it relates to the long interval estimates of time perception measured (described below).

#### Long Interval Measures of Timing

Directly after completing both the delay discounting task and the interval bisection task, participants answered two questions assessing their perception of time using longer time intervals (as opposed to the short time intervals examined by the interval bisection task). The first question was, "How quickly has time seemed to pass since you first arrived and signed the informed consent?" with "time flew" (1), and "time dragged" (5) as anchors. The second question was an open-ended question, "How many minutes would you estimate have elapsed since you signed the informed consent?" Hereafter the first timing question will be referred to as 'scale long task' and the second timing question will be referred to as 'numerical estimate long task.' Following these assessments of time perception, participants answered several basic demographic questions (e.g., age, sex).

### Data Analysis

Of the 45 individuals that participated, data for 2 were not considered due to nonsystematic discounting [47]. Delay discounting data were considered systematic and used if indifference points did not increase across consecutive delays by more than 20% of the larger later reward—and thus assumes a monotonically decreasing function [47]. To evaluate impulsive decision-making within the delay discounting task, two measures of impulsivity were assessed: *k* values and Area Under the Curve (AUC). To examine *k* values (which represent degree of delay discounting), the following widely employed simple hyperbola was used [10]:

V=A/(1+kD) (1)

where V is the subjective value of the outcome (i.e., the indifference point, or the value at which the immediate and delayed options are of equal subjective value), A is the amount of the delayed reward, D is the delay to receipt of the reward, and *k* is the degree to which the value of the reward decreases with delay. The values of A and D are predetermined based on the values used within the research context (e.g., if the delayed reward used is 100 dollars, then the numerator is 100).

Eq 1 was fit to the median indifference points for the natural condition and the built condition using nonlinear regression (GraphPad Prism®). Fitting a curve to the indifference points across conditions enables visual assessment of how well the formula describes the data, as well as visual comparison of impulsivity across natural and built conditions. Resulting *k* parameter values serve as a comparison across groups, and offer a measure of impulsive decision-making. High *k* values represent more impulsive decision-making.

Area Under the Curve was computed as an atheoretical measure of delay discounting. In order to calculate the AUC, the delays and indifference points are normalized. Then the area underneath the curve is calculated using the equation *x*2 −*x*1[(*y*1 + *y*2)/2], where x2 and x1 represent successive delays and y1 and y2 represent the indifference points associated with those delays [48]. These values are then summed, resulting in AUC calculations between 0 and 1, with higher numbers representing more self-control.

For time perception, we were interested in two primary questions related to a potential relation between viewing natural scenes and impulsivity. First, did viewing natural scenes alter time perception relative to built scenes? To examine this, two-tailed *t*—tests were conducted to assess the effects of condition (natural versus built) on the two long interval time estimate questions. Second, is time perception associated with impulsivity? To test this, correlations between impulsivity and long interval time estimates were computed. Values for AUC and *k* were not normally distributed [49] so statistical analyses presented below do not assume Gaussian distributions (i.e., all statistical analyses presented with impulsivity measures are Mann Whitney *t*-tests or Spearman correlations).

## Results

Table 1 presents demographic information for participants in the natural and built conditions. Of the 43 participants, 17 were male and 26 were female. The mean age was 22.5 years (SD = 5.3). *T*-tests comparing demographic make-up across the natural and built conditions indicated no significant differences across groups for these variables—age (*t* [41] = .355, *p* = .725), sex (*t* [41] = 1.048, *p* = .301), or ethnicity (*t* [38] = .555, *p* = .582; note that not all participants chose to report ethnicity).

**Table 1:** Demographic Information for Natural and Built Conditions.

	Condition	
	Natural	Built
Proportion Caucasian	19/22	16/21
Proportion Male	7/22	10/21
Mean Age (SD)	22.82 (6.48)	22.24 (3.85)

### Main effects of viewing nature scenes: Impulsivity Measurements

**Fig 1:**
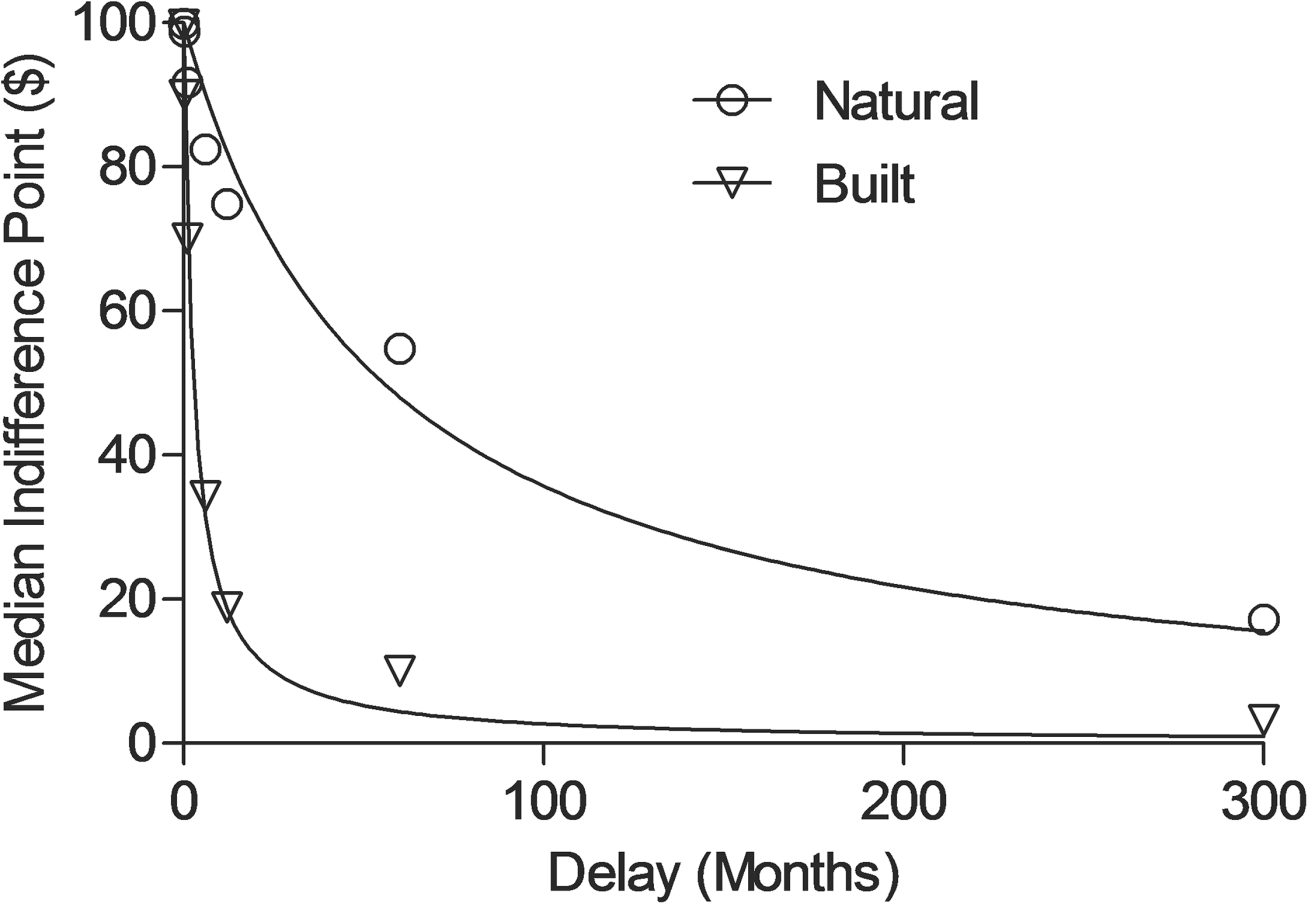
Indifference points as a function of delay for the natural and built conditions. Median indifference points as a function of delay (months) for natural (circles) and built (triangles) conditions. Lines show the best fit of Eq 1 to the median indifference points.

Table 2 displays all variables analyzed in the present experiment. Fig 1 displays the median indifference points for the natural (n = 22) and built (n = 21) conditions. Median indifference points decreased as a function of delay in each condition. Participants exposed to scenes of natural environments exhibited less impulsivity relative to those exposed to built environments (i.e., higher indifference points were revealed in the natural condition). Eq 1 provided good fits to the median indifference points for the natural (R^2^ = .96; *k* = .018) and built (R^2^ = .99; *k* = .360), conditions as well as the indifference points of individual participants (Natural Mdn R^2^ = .92; Built Mdn R^2^ = .96). Median *k* values were .015 and .477 for the natural and built conditions, respectively. Two tailed t-tests confirmed that participants who viewed natural environments showed less impulsivity (lower *k* values) than participants viewing built environments (Mann-Whitney t-test; U = 131, *p* = .016, Cliff's Delta = .43). In addition to the *k* values obtained by using Eq 1, the same analyses were applied using delay discounting models proposed by Rachlin [50], Green and Myerson [51], and Takahashi [52], and yielded similar conclusions.

**Table 2:** Timing and Impulsivity Measures for the Natural and Built Conditions.

	Natural Condition						
	P #	Average Proportion Long Response	Scale Long Task -Time "flew" (1) or "dragged" (5)	Numerical Estimate Task (Estimation of Session Length in Minutes)	Actual Session Time (Minutes)	AUC	*k*
	1	0.365	5	45	22.25	0.995	0.000
	2	0.651	4	30	23.65	0.689	0.003
	3	0.492	5	15	23.58	0.682	0.004
	4	0.460	3	30	22.14	0.190	0.098
	5	0.587	3	35	26.13	0.019	1.957
	6	0.492	3	30	26.3	0.996	0.000
	7	0.619	3	15	27.63	0.795	0.001
	8	0.683	4	35	24.35	0.780	0.003
	9	0.540	2	35	24.5	0.048	0.485
	10	0.651	3	40	23.78	0.284	0.172
	11	0.460	2	30	23.45	0.718	0.003
	12	0.429	4	30	23.43	0.350	0.022
	13	0.508	4	35	23.53	0.217	0.185
	14	0.635	4	20	22.24	0.070	1.896
	15	0.476	3	25	21.69	0.547	0.009
	16	0.587	3	30	26.31	0.747	0.002
	17	0.524	4	40	28.18	0.093	0.805
	18	0.476	3	40	21.76	0.281	0.050
	19	0.556	5	30	23.49	0.429	0.017
	20	0.524	3	30	23.66	0.044	0.311
	21	0.317	2	40	22.36	0.467	0.011
	22	0.429	4	35	26.13	0.427	0.013
Mean		0.52	3.45	31.59	24.12	0.45	0.27
SD		0.09	0.91	7.77	1.89	0.31	0.57
	Built Condition						
	P #	Average Proportion Long Response	Scale Long Task -Time "flew" (1) or "dragged" (5)	Numerical Estimate (Estimation of Session Length in Minutes)	Actual Session Time (Minutes)	AUC	*k*
	23	0.383	5	15	22.38	0.451	0.101
	24	0.432	3	20	21.59	0.015	5.738
	25	0.573	2	20	24.21	0.092	0.777
	26	0.547	4	25	22.16	0.965	0.000
	27	0.519	2	22	24.34	0.011	3.267
	28	0.432	4	25	28.41	0.021	1.799
	29	0.534	3	25	22.66	0.138	0.480
	30	0.421	3	27	22.3	0.022	2.222
	31	0.529	3	25	28.33	0.354	0.027
	32	0.472	3	25	23.45	0.101	1.815
	33	0.468	3	25	21.84	0.027	0.477
	34	0.618	4	35	23.62	0.921	0.001
	35	0.442	2	20	22.3	0.052	0.386
	36	0.625	3	40	24.33	0.092	0.283
	37	0.716	5	28	28.26	0.202	0.138
	38	0.571	2	20	21.73	0.044	0.528
	39	0.634	3	45	21.97	0.153	0.270
	40	0.335	3	30	21.66	0.125	0.925
	41	0.553	2	25	22.26	0.226	0.026
	42	0.512	3	20	24.42	0.999	0.000
	43	0.577	3	30	28.2	0.053	0.706
Mean		0.52	3.10	26.05	23.83	0.24	0.95
SD		0.09	0.89	7.07	2.41	0.32	1.40

**Fig 2:**
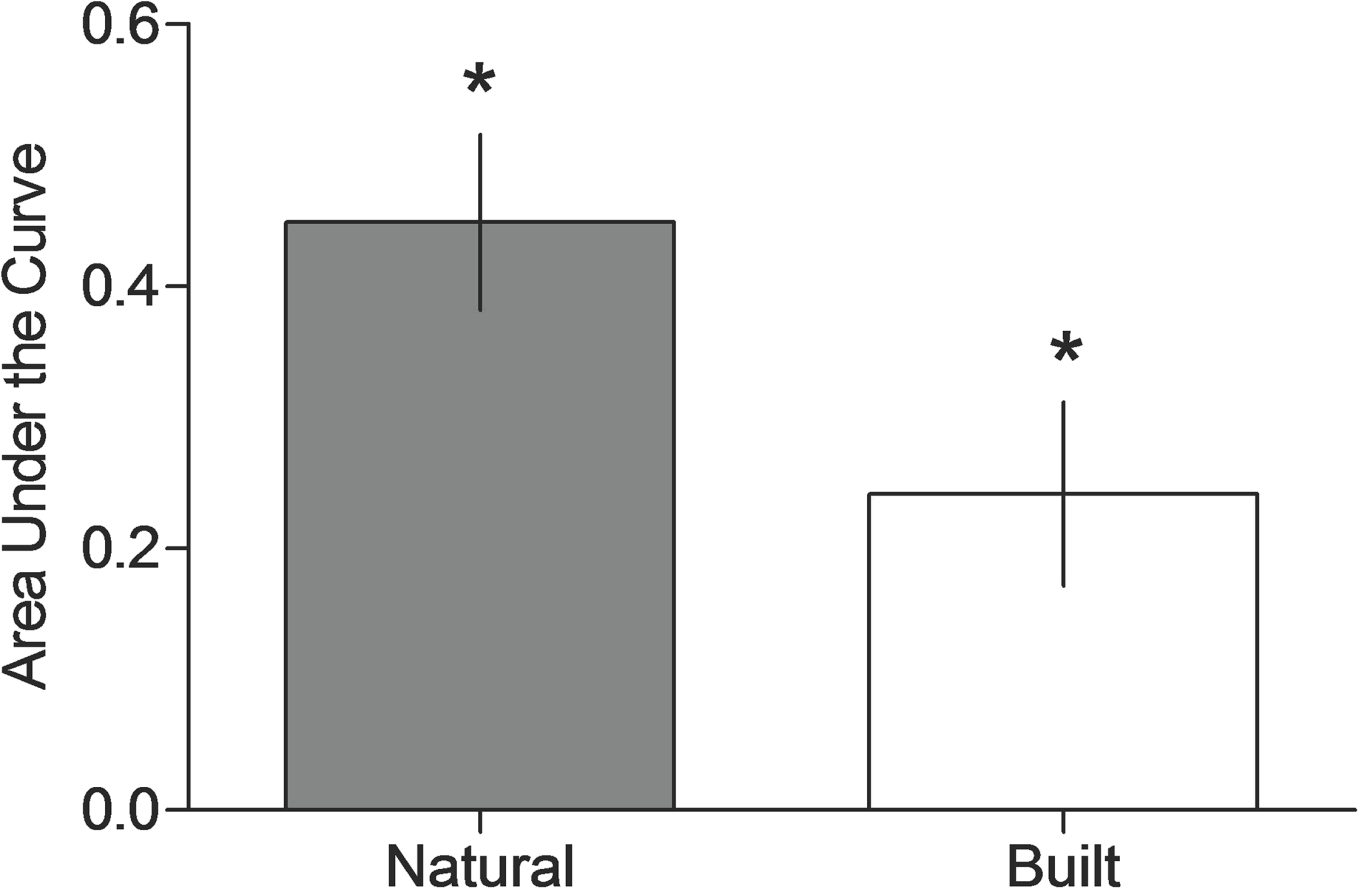
Area Under the Curve for the natural and built conditions. Mean AUC for the natural (open bar) and built (filled bar) conditions. Vertical lines represent the standard error of the mean.

Fig 2 displays the mean AUC for the natural (n = 22) and built (n = 21) conditions. As with *k* values, participants exposed to scenes of natural environments exhibited less impulsivity relative to those exposed to built environments (i.e., higher AUC revealed in the natural condition). The AUC indicated greater levels of self-control in the natural relative to built condition (U = 136, *p* = .0217, Cliff's Delta = .41). As would be expected, a strong and significant negative correlation between *k* and AUC was observed (Spearman correlation, r = -.95, n = 43, *p* < .0001).

### Main effects of viewing nature scenes: Time Perception Measurements

#### Session Time

To verify that there were no differences in session length across conditions, we calculated actual session times. Session times were nearly identical across natural and built conditions (see Table 2), and as such were not significantly different (two-tailed t test, *t* [41] = .44, *p* = .67). Thus, any differences in long interval time estimates cannot be attributed to mean differences in actual time between sessions. Again, all actual time and perceived time measures are presented in Table 2, including the null interval bisection findings.

#### Numerical estimate long task

**Fig 3:**
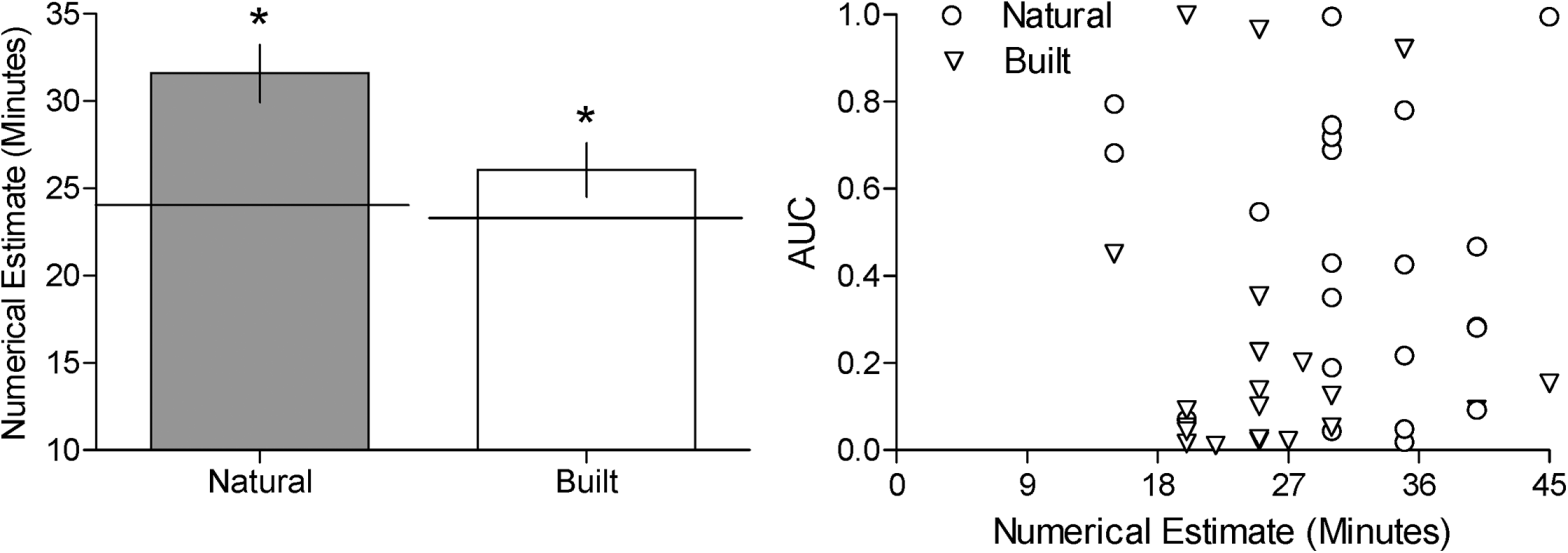
Estimation of session length in minutes for the natural and built conditions (left panel) and scatter plot of AUC as a function of Numerical Estimate (right panel). Mean estimate of session length in minutes for the natural (filled bar) and built (open bar) conditions (left panel). Horizontal lines represents the actual session length mean rounded to the nearest minute for both the natural and built conditions. Scatter plot of AUC as a function of Numerical Estimate in Minutes (right panel)—circles and triangles represent participant responses in the natural and built conditions, respectively. Asterisk represent significant differences in time estimation across natural and built conditions. Vertical lines represent the standard error of the mean.

Although in both conditions participants experienced almost identical amounts of time passing in reality, those in the Natural condition estimated significantly more minutes had elapsed than those in the Built condition (Fig 3—left panel; *t* [41] = 2.44, *p* = .019, *d* = .713). This numerical estimate long task was significantly different from the actual elapsed time in the Natural condition (paired *t*-test, t [21] = 4.60, *p* = .0002, *d* = .962), but not the Built condition (paired *t*-test, t [20] = 1.619, *p* = .1212). This time perception measure, however, was not significantly correlated with impulsivity measures (AUC; Fig 3—right panel; Spearman correlation, r = .1323, n = 43, *p* = .398; *k*; r = -.153, n = 43; *p* = .328).

#### Scale long task

**Fig 4:**
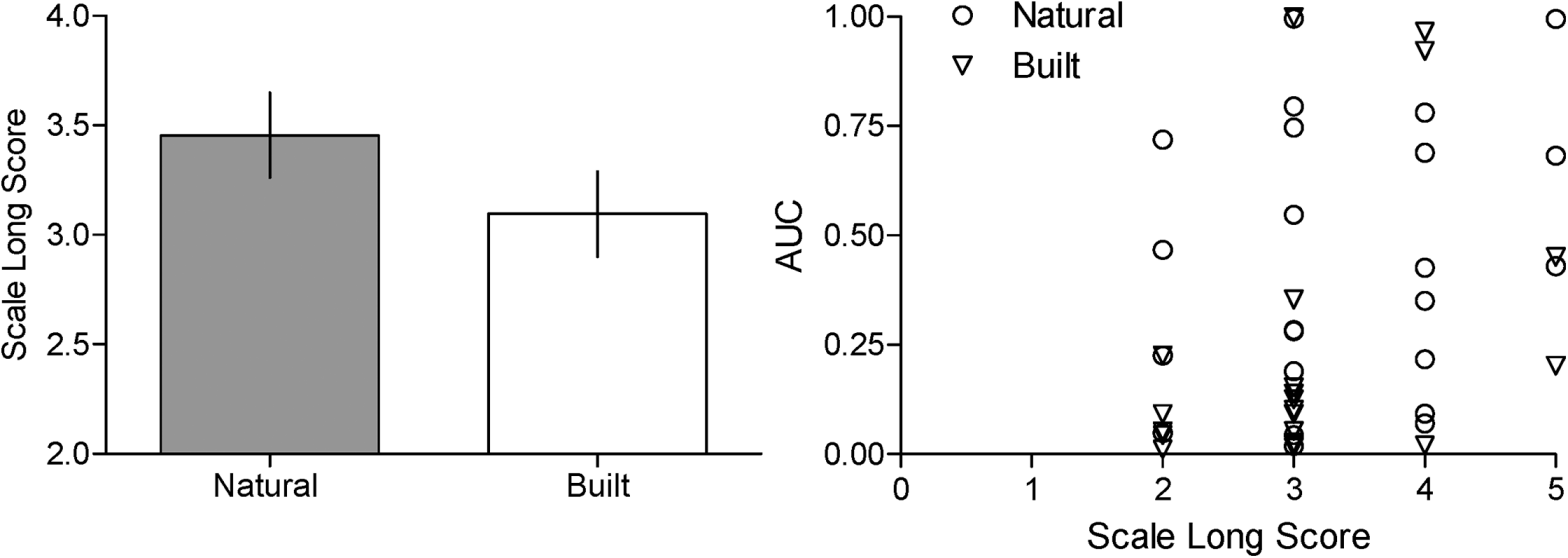
Score on the scale long task for the natural and built conditions (left panel) and scatter plot of AUC as a function of Scale Long Score (right panel). Mean score on the 'scale long task' for the natural (filled bar) and built (open bar) conditions (left panel). Vertical lines represent the standard error of the mean. Scatter plot of AUC as a function of the Scale Long Score (right panel)—circles and triangles represent participant responses in the natural and built conditions, respectively.

Contrary to the numerical estimate long task, the scale long task was found to be significantly correlated with impulsivity as measured by AUC (Fig 4—right panel; Spearman correlation, r = .353, n = 43, *p* = .020), with it only trending towards significance with *k*, however (*k*; r = -.277, n = 43, *p* = .072). Fig 4 shows that, consistent with the effect of condition on the numerical estimate long task, those in the Natural condition reported that time had 'dragged' more so than those in the Built condition (right panel) on the scale long task (time flew [1] versus time dragged [5]); however, this difference was not statistically significant (*t* [41] = 1.31, *p* = .198).

## Discussion

Several notable outcomes emerged from the present study in which we evaluated the effects of visual exposure to natural versus built stimuli on impulsivity and time perception. These results (I) validate previous research showing that exposure to natural environments results in less impulsivity (i.e., greater self-control), (II) show novel evidence that exposure to natural environments can lengthen perception of time, and (III) show evidence for the relationship between time perception and impulsivity. Below, we explore each of these in turn, while discussing similarities and differences between this study and previous research.

### Natural Environments and Impulsivity

Consistent with previous findings [2,35] exposure to natural environments resulted in greater self-control relative to exposure to built environments. Given that this has been demonstrated using different natural and built stimuli as well as real world natural and built exposure across various impulsivity tasks [2,35], this effect appears to be robust. This line of research adds to the growing body of evidence highlighting the beneficial aspects of natural environments for humans. Exposure to natural environments reduces stress, increases happiness, improves mood and restores attention [44, 53–56]. The present study confirms that apart from cognitive and mood influences, exposure to natural environments may also improve human decision-making.

These results are particularly important given the potentially sweeping implications of nature’s effect on impulsivity. Reducing impulsivity in one realm has been shown to influence impulsivity in other realms [30], suggesting similar underlying processes at work. Thus, the present results have implications for global reductions in impulsive decision-making that may apply not only to disorders associated with impulse control, but also to our everyday decision-making–including those in the environmental realm (e.g., the choice to take public transportation with an increased delay but reduced emissions, rather than driving a private car with a reduced delay but more emissions, [2, 11, 57]).

### Natural Environments and Time Perception

Time perception is malleable–it psychologically speeds up and slows down based on various cues [58–60]. The present study provides evidence that part of this variability in time perception is due to the presence or absence of natural environments. Merely viewing natural environments can lengthen time perception: people who viewed natural environments reported longer time estimates than those who viewed built environments.

This effect was strongest (and statistically significant) for the more *objective* measurement of the passage of long intervals of time (the estimate involving minutes passed and not scale ratings of perceived time speed). This makes sense in that the scale long measure was not *anchored* with the actual time passed, and thus it is less clear what a low or high score might mean in relation to actual time. In contrast, the numerical estimate long task in minutes can be directly compared to the actual amount of time, and thus is a more precisely anchored measurement, in this context, of the “lengthening” of time.

### Natural Environments, Time Perception, and Impulsivity

Viewing natural scenes influenced both time perception and impulsivity, although this was manifested in different ways across the two separate long interval time perception measures. First, the ‘scale long task’ measurement, while showing a weaker relation to the experimental manipulation than the ‘numerical estimate long task’ measurement, showed a stronger (and significant) relationship to impulsivity. It is possible that impulsivity is more related to long interval scale measurements of time perception because those scale measurements are more subjective in nature. The 'scale long task' measurement is a measurement of what time “feels” like, unanchored by actual time, and it could be that that feeling is more tied to impulsivity than a more objective measurement such as the ‘numerical estimate long task’.

Taken in total, then, we have (a) a time-impulsivity effect for a scale measurement of time perception (scale long task), and (b) a time-condition effect for the minutes passed estimate measurement of time perception (numerical estimate long task). Further, there was no effect of short interval time perception (measured by temporal bisection) in relation to impulsivity or condition. Baumann and Odum [13] showed that although some measures of time (an interval bisection task) were weakly correlated with impulsivity in a delay discounting task, others were not (Zimbardo Time Perspective Inventory), and thus the present results are not necessarily surprising. Although we did not show that time perception as measured by an interval bisection task was correlated with impulsivity in the present experiment, we used shorter stimulus durations than those used previously [13] making direct comparisons difficult. It is possible that longer as opposed to shorter stimulus durations in an interval bisection task correlate more closely to the relatively longer time considerations presented in delay discounting tasks (i.e., days to years). It should also be noted that because the numerical estimate long task (estimate in minutes) by necessity followed the delay discounting task, differences in time perception may be influenced by the natural-built manipulation and choices of larger versus smaller rewards, rather than an intrinsic relation between timing and impulsivity. More research on this topic is warranted.

Some evidence from other timing studies shows that those who perceive time to pass more slowly tend to be *more impulsive* ([13], see [40] for a review on time perception and impulsivity). These studies, however, have largely been conducted with timing discrimination tasks at very short intervals, measuring fine temporal perception within the milliseconds to seconds range [40]. Recall that in the present study, we found no differences in time perception across natural and built conditions at very short timing intervals (i.e., milliseconds to seconds). It is possible that lengthened time perception of longer intervals (i.e., minutes or longer) with exposure to natural environments, may have different effects on impulsive decision-making and may represent something akin to an expanded perception of time in which waiting for delayed consequences is less aversive. Another interpretation is that lengthened time perception of longer intervals enables individuals to bridge the gap between the present behavior and future consequences, or possibly represents greater future foresight [38]. These results converge with other evidence suggesting that the focus on temporal domains alters delay discounting [37], although the direct relation between millisecond to second time perception and longer durations is still unclear. More evidence is needed to draw firm conclusions on the relation between exposure to natural environments, time perception, and impulsivity, and the relations between short and long interval timing mechanisms.

Considering the consistent pattern of previous research on the relationship between time perception and impulsivity, along with the present results, we suspect that there is a connection between time perception and impulsivity, but that the mechanism driving differences in impulsivity across environmental conditions is more complex than simply time perception. For example, as attention is restored by visual exposure to natural relative to built environments [44] and increased attention is also related to decreased impulsivity [61], it is possible that attention and/or arousal—which we did not measure in the present study—combined with temporal perception, also influences differences in impulsivity observed across natural and built scenes. More research is necessary before drawing a firm conclusion about the underlying mechanisms driving differences in impulsivity with exposure to natural versus built environments, but the present study adds important evidence to this discussion.

## Conclusion

This study provides evidence for the importance of better understanding the role of time perception in discounting of future outcomes, and how this can differ by exposure to natural or built environments. Prior research has demonstrated that those who discount the future steeply in one area (e.g., money, health), discount the future steeply in other areas (e.g., environmental), suggesting similar underlying processes governing these decisions [11,17]. Thus, identifying techniques which increase future valuation (i.e., less impulsive decisions) of outcomes like money, as in the present study, may also serve to increase future valuation of other outcomes such as health and environmental outcomes [30]. Such global changes in decision-making may be driven in part by changes in time perception, and this may be influenced by environments in which we spend our time. Continued research in this vein may ultimately provide a foundation for understanding how we might promote healthy, future-oriented and sustainable decision-making that will benefit ourselves and our ecosystems.

## References

[1] Chivian E, Bernstein A. Preface In Chivian E, Bernstein A (editors), Sustaining life: How human health depends on biodiversity (XI-XIII). 2008; New York, NY: Oxford University Press, Inc.

[2] van der Wal AJ, Schade HM, Krabbendam L, van Vugt M. Do natural landscapes reduce future discounting in humans? P Roy Soc B-Biol Sci. 2013; 280: 20132295. 10.1098/rspb.2013.2295 PMC3826228 24197412

[3] Anderson K, Bows A. Beyond ‘dangerous’ climate change: Emission scenarios for a new world. Philos T R Soc A. 2011; 369 (1934): 20–44. 10.1098/rsta.2010.0290 21115511

[4] Boden T, Blasing TJ. Record High (2010) Global Carbon Dioxide Emissions. From Carbon Dioxide Information Analysis Center. 2011; Available: http://cdiac.ornl.gov/trends/emis/perlim_2009_2010_estimates.html.

[5] Pimm SL, Jenkins CN, Abell R, Brooks TM, Gittleman JL, Joppa LN, et al The biodiversity of species and their rates of extinction, distribution, and protection. Science. 2014; 344: 1246752 10.1126/science.1246752 24876501

[6] Barnosky AD, Matzke N, Tomiya S, Wogan GOU, Swartz B, Quental CM, et al Has the earth's sixth mass extinction already arrived? Nature. 2011; 471: 51–57. 10.1038/nature09678 21368823

[7] Odum AL. Delay Discounting: I'm a K: you're a K. J Exp Anal Behav. 2011; 96: 427–439. 10.1901/jeab.2011.96-423 22084499 PMC3213005

[8] National Institutes on Drug Abuse. Trends & Statistics. 2014; Available: http://www.drugabuse.gov/related-topics/trends-statistics. Accessed 2014 Aug 16.

[9] Centers for Disease Control and Prevention. Adult obesity facts. 2014; Available: http://www.cdc.gov/obesity/data/adult.html. Accessed 2014 Aug 16.

[10] Mazur JE. An adjusting procedure for studying delayed reinforcement In: Commons ML, Mazur JE, Nevin JA, Rachlin H. (editors), Quantitative Analyses of Behavior: Vol. 5: The Effect of Delay and of Intervening Events on Reinforcement Value. 1987; Earlbaum, Hillsdale, NJ, pp. 55–73.

[11] Hardisty DJ, Weber EU. Discounting future green: money versus the environment. J Exp Psychol Gen. 2009; 138: 329–340. 10.1037/a0016433 19653793

[12] Hepburn C, Duncan S, Papachristodoulou A. Behavioural economics, hyperbolic discounting, and environmental policy. Environ Res Econ. 2010; 46: 189–206.

[13] Baumann A, Odum AL. Impulsivity, risk taking and timing. Behav Process. 2012; 90: 408–414. 10.1016/j.beproc.2012.04.005 PMC3897391 22542458

[14] de Wit H. Impulsivity as a determinant and consequence of drug use: a review of underlying processes. Addict Biol. 2008; 14: 22–31. 10.1111/j.1369-1600.2008.00129.x 18855805 PMC3640851

[15] Kaplan BA, Reed DD, McKerchar TL. Using a visual analogue scale to assess delay, social, and probability discounting of an environmental loss. Psychol Rec. 2014; 64: 261–269.

[16] Madden GJ, Bickel WK. Introduction In: Madden GJ, Bickel WK (editors), Impulsivity: The Behavioral and Neurological Science of Discounting. 2010; APA Books, Washington, DC, pp. 3–8.

[17] Odum AL. Delay discounting: Trait variable? Behav Process. 2011; 87: 1–9. 10.1016/j.beproc.2011.02.007 PMC3266171 21385637

[18] Reed SC, Levin FR, Evans SM. Alcohol increases impulsivity and abuse liability in heavy drinking women. Exp Clin Psychopharm. 2012; 20: 454–65. 10.1037/a0029087 PMC3598581 23066857

[19] Friedel JE, Dehart WB, Madden GJ, Odum AL. Impulsivity and cigarette smoking: discounting of monetary and consumable outcomes in current and non-smokers. Psychopharmacology. 2014; 231 (23): 4517–4526. 10.1007/s00213-014-3597-z 24819731 PMC4221621

[20] Sheffer CE, Christensen DR, Landes R, Carter LP, Jackson L, Bickel WK. Delay discounting rates: a strong prognostic indicator of smoking relapse. Addict Behav. 2014; 39: 1682–9. 10.1016/j.addbeh.2014.04.019 24878037 PMC4316833

[21] Weller RE, Cook EW III, Avsar KB, Cox JE. Obese women show greater delay discounting than healthy-weight women. Appetite. 2008; 51: 563–569. 10.1016/j.appet.2008.04.010 18513828

[22] Dixon MR, Marley J, Jacobs EA. Delay discounting by pathological gamblers. J Appl Behav Anal. 2003; 36: 449–458. 14768665 10.1901/jaba.2003.36-449 PMC1284461

[23] Chabris CF, Laibson D, Morris CL, Schuldt JP, Taubinsky D. Individual laboratory-measured discount rates predict field behavior. J Risk Uncertainty. 2008; 37: 237–269. 10.1007/s11166-008-9053-x PMC2676104 19412359

[24] Lagorio CH, Madden GJ. Delay discounting of real and hypothetical rewards III: steady-state assessments, forced-choice trials, and all real rewards. Behav Process. 2005; 69: 173–87. 10.1016/j.beproc.2005.02.003 15845306

[25] Anokhin AP, Golosheykin S, Grant JD, Heath AC. Heritability of delay discounting in adolescence: A longitudinal twin study. Behav Genet. 2011; 41: 175–183. 10.1007/s10519-010-9384-7 20700643 PMC3036802

[26] McClure SM, Laibson DI, Loewenstein G, Cohen JD. Separate neural systems value immediate and delayed monetary rewards. Science. 2004; 306 (5695): 503–507. 15486304 10.1126/science.1100907

[27] Peper JS, Mandl RC, Braams BR, de Water E, Heijboer AC, Koolschijn PC, et al Delay Discounting and Frontostriatal Fiber Tracts: A Combined DTI and MTR Study on Impulsive Choices in Healthy Young Adults. Cereb Cortex. 2013; 23: 1695–1702. 10.1093/cercor/bhs163 22693341 PMC3673180

[28] Dixon MR, Jacobs EA, Sanders S. Contextual control of delay discounting by pathological gamblers. J Appl Behav Anal. 2006; 39: 413–422. 17236338 10.1901/jaba.2006.173-05 PMC1702333

[29] Gilman JM, Curran MT, Calderon V, Stoeckel LE, Evins AE. Impulsive social influence increases impulsive choices on a temporal discounting task in young adults. PLoS One. 2014; e101570 10.1371/journal.pone.0101570 24988440 PMC4079280

[30] Black AC, Rosen MI. A money management-based substance use treatment increases valuation of future rewards. Addict Behav. 2011; 36: 125–128. 10.1016/j.addbeh.2010.08.014 20826055 PMC2981645

[31] Daniel TO, Stanton CM, Epstein LH. The future is now: Reducing impulsivity and energy intake using episodic future thinking. Psychol Sci. 2013; 24: 2339–2342. 10.1177/0956797613488780 24022653 PMC4049444

[32] Johnson PS, Herrmann ES, Johnson MW. Opportunity costs of reward delays and the discounting of hypothetical money and cigarettes. J Exp Anal Behav. 2015; 103: 87–107. 10.1002/jeab.110 25388973 PMC4428151

[33] Rung JM, Young ME. Learning to wait for more likely or just more: Greater tolerance to delays of reward with increasingly longer delays. J Exp Anal Behav. 2015; 103: 108–124. 10.1002/jeab.132 25641081 PMC9211002

[34] Koffarnus MN, Jarmolowicz DP, Mueller ET, Bickel WK. Changing delay -discounting in the light of the competing neurobehavioral decision systems theory: A review. J Exp Anal Behav. 2013; 99: 32–57. 10.1002/jeab.2 23344987 PMC3917566

[35] Berry MS, Sweeney MM, Morath J, Odum AL, Jordan KE. The nature of impulsivity: Visual exposure to natural environments decreases impulsive decision-making in a delay discounting task. PLoS ONE 2014; 9(5): e97915 10.1371/journal.pone.0097915 24841421 PMC4026519

[36] Rudd M, Vohs KD, Aaker J. Awe expands people's perception of time, alters decision making, and enhances well-being. Psychol Sci. 2012; 23: 1130–1136. 10.1177/0956797612438731 22886132

[37] Radu PT, Yi R, Bickel WK, Gross JJ, McClure SM. A mechanism for reducing delay discounting by altering temporal attention. J Exp Anal Behav. 2011; 96: 363–85. 10.1901/jeab.2011.96-363 22084496 PMC3213002

[38] Peters J, Büchel C. Episodic Future Thinking Reduces Reward Delay Discounting through an Enhancement of Prefrontal-Mediotemporal Interactions. Neuron. 2010; 66: 138–148. 10.1016/j.neuron.2010.03.026 20399735

[39] Ungemach C, Stewart N, Reimers S. How incidental values from the environment affect decisions about money, risk, and delay. Psychol Sci. 2011; 22: 253–260. 10.1177/0956797610396225 21228134 PMC5496680

[40] Rubia K, Halari R, Christakou A, Taylor E. Impulsiveness as a timing disturbance: neurocognitive abnormalities in attention-deficit hyperactivity disorder during temporal processes and normalization with methylphenidate. Philos Trans R Soc Lond B Biol Sci. 2009; 364:1919–1931. 10.1098/rstb.2009.0014 19487194 PMC2685816

[41] Wittmann M, Leland D, Churan J, Paulus MP. Impaired time perception and motor timing in stimulant-dependent subjects. Drug Alcohol Depen. 2007; 90:183–192. 10.1016/j.drugalcdep.2007.03.005 PMC1997301 17434690

[42] Wittman M, Simmons AN, Flagan T, Lane SD, Wackermann J, Paulus MP. Neural substrates of time perception and impulsivity. Brain Res. 2011; 1406:43–58. 10.1016/j.brainres.2011.06.048 21763642 PMC3145046

[43] Wittmann M, Paulus MP. Temporal horizons in decision making. J. Neurosci. Psychol Econ. 2009; 2:1–11.

[44] Berto R. Exposure to restorative environments helps restore attention capacity. J Environ Psychol. 2005; 25: 249–259.

[45] Kangas BD, Berry MS, Cassidy RN, Dallery J, Vaidya M, Hackenberg TD. Concurrent Performance in a 3-Alternative Choice Situation: Response Allocation in Rock/Paper/Scissors Game. Behav Process. 2009; 82: 164–172. 10.1016/j.beproc.2009.06.004 19555744

[46] Rodzon K, Berry MS, Odum AL. Within-subject comparison of degree of delay discounting using titrating and fixed sequence procedures. Behav Process. 2011; 86: 164–167. 10.1016/j.beproc.2010.09.007 PMC3919556 20932882

[47] Johnson MW, Bickel WK. An algorithm for identifying nonsystematic delay-discounting data. Exp Clin Psychopharm. 2008;16: 264–274. 10.1037/1064-1297.16.3.264 PMC2765051 18540786

[48] Myerson J, Green L, Warusawitharana M. Area under the curve as a measure of discounting. J Exp Anal Behav. 2001; 76: 235–243. 11599641 10.1901/jeab.2001.76-235 PMC1284836

[49] Rachlin H, Raineri A, Cross D. Subjective probability and delay. J Exp Anal Behav. 1991; 2: 233–244. 10.1901/jeab.1991.55-233 PMC1323057 2037827

[50] Rachlin H. Notes on discounting. J Exp Anal Behav. 2006; 85: 425–435. 16776060 10.1901/jeab.2006.85-05 PMC1459845

[51] Green L, Myerson J. A discounting framework for choice with delayed and probabilistic rewards. Psychol Bull. 2004; 130: 769–792. 15367080 10.1037/0033-2909.130.5.769 PMC1382186

[52] Takahashi T. A comparison of intertemporal choices for oneself versus someone else based on Tsallis’ statistics. Physica A. 2007; 385: 637–644.

[53] Ward Thompson C, Roe J, Aspinall P, Mitchell R, Clow A, Miller D. More green space is linked to less stress in deprived communities: Evidence from salivary cortisol patterns. Landscape Urban Plan. 2012; 105(3): 221–229.

[54] White M, Alcock I, Wheeler B, Depledge MH. Would you be happier living in a greener urban area? A fixed-effects analysis of panel data. Psychol Sci. 2013; 24: 920–928. 10.1177/0956797612464659 23613211

[55] Ulrich RS. Natural versus urban scenes: some psychophysiological effects. Environ Behav. 1981; 13: 523–556.

[56] Bowler DE, Buyung-Ali LM, Knight TM, Pullin AS. A systematic review of evidence for the added benefits to health of exposure to natural environments. BMC Public Health. 2010; 10: 456–465. 10.1186/1471-2458-10-456 20684754 PMC2924288

[57] Arbuthnott KD. Taking the long view: Environmental sustainability and delay of gratification. Anal of Soc Issues Public Policy. 2010; 10: 4–22.

[58] Conway LG III. Social contagion of time perception. J Exp Soc Psychol. 2004; 40 (1): 113–120.

[59] Droit-Volet S, Fayolle SL, Gil S (2011) Emotion and time perception: Effects of film-induced mood. Front in Integr Neurosci. 2011 5: 33 10.3389/fnint.2011.00033. PMC3152725 21886610

[60] Droit-Volet S, Meck WH. How emotions colour our perception of time. Trends Cogn Sci. 2007; 11(12): 504–513. 18023604 10.1016/j.tics.2007.09.008

[61] Barkley RA, Edwards G, Laneri M, Fletcher K, Metevia L. Executive functioning, temporal discounting, and sense of time in adolescents with attention deficit hyperactivity disorder (ADHD) and oppositional defiant disorder (ODD). J Abnorm Child Psych. 2001; 6: 541–556. 10.1023/a:1012233310098 11761287

